# The Role of Microglia in Perioperative Neurocognitive Disorders

**DOI:** 10.3389/fncel.2020.00261

**Published:** 2020-08-18

**Authors:** Wenguo Fan, Lijia Mai, Xiao Zhu, Fang Huang, Hongwen He

**Affiliations:** ^1^Department of Anesthesiology, Guanghua School of Stomatology, Hospital of Stomatology, Sun Yat-sen University, Guangzhou, China; ^2^Guangdong Provincial Key Laboratory of Stomatology, Guangzhou, China; ^3^The Marine Biomedical Research Institute, Guangdong Medical University, Zhanjiang, China

**Keywords:** perioperative neurocognitive disorder, microglia, neuroinflammation, anesthesia, surgery

## Abstract

Perioperative neurocognitive disorder (PND) is a common phenomenon associated with anesthesia and surgery and has been frequently described in the elderly and susceptible individuals. Microglia, which are the brain’s major resident immune cells, play critical roles in maintaining neuronal homeostasis and synaptic plasticity. Accumulating evidence suggests microglial dysfunction occurring after anesthesia and surgery might perturb neuronal function and induce PND. This review aims to provide an overview of the involvement of microglia in PND to date. Possible cellular and molecular mechanisms regarding the connection between microglial activation and PND are discussed.

## Introduction

Microglia are a type of neuroglia occurring in the central nervous system (CNS) and can be defined as tissue-resident macrophages (Greter et al., 2015; Chowen and Garcia-Segura, 2020). They play important roles in the sustainment of normal physiological functions of CNS. A large amount of evidence indicates that microglia are involved in neuroinflammation as their activation and has been associated with many neurological disorders such as Alzheimer’s disease (AD), neuropsychiatric disorders (Nayak et al., 2014).

Disordered neurocognitive function after surgery and anesthesia is a heterogeneous set of conditions, which includes any form of the acute event (postoperative delirium) and cognitive decline diagnosed up to 30 days after the procedure (delayed neurocognitive recovery) and up to 12 months (postoperative neurocognitive disorder, POCD; Evered et al., 2018; Safavynia and Goldstein, 2018). Previously, all forms of the impairment were called POCD, but more recently, perioperative neurocognitive disorders (PND) are recommended to be used as an overarching term for cognitive impairment identified in the perioperative period (Evered et al., 2018). PND is characterized as an acute or durable disturbance of cognitive functions including awareness, memory, attention, information processing, and cognitive flexibility (Hovens et al., 2012). The incidence of PND ranges from 8.9% to 46.1% depending on the study and type of surgery (Androsova et al., 2015). It occurs commonly in older patients (Monk et al., 2008; Evered et al., 2018). The pathogenesis of PND is multifaceted, which might be associated with anesthesia, tissue damage, neuroinflammation, surgical stress, psychological stress, and so on. In human studies, patients who develop PND showed cerebrospinal fluid (CSF) and plasma pro-inflammatory cytokines increases after anesthesia and surgery (Ji et al., 2013; Hirsch et al., 2016; Yuan et al., 2020). The more pronounced changes in CSF cytokines compared to plasma for several cytokines (MCP, MIP-1α, MIP-1β) provide evidence for substantial inflammatory activity in the CNS (Hirsch et al., 2016). Since microglia are the macrophages of the CNS and play critical roles in neuroinflammatory disease (West et al., 2019), the significant alterations in some cytokines in CSF from patients indicate that microglia may be involved in PND in human (Helmy et al., 1999; Bromander et al., 2012; Hirsch et al., 2016; Yuan et al., 2020). Mounting evidence from animal studies suggests that microglia, like immune cells, are activated in the CNS and implicated in neuroinflammation and PND. This review aims to give an overview of the involvement of microglia in PND to date. Possible mechanisms regarding the connection between microglia and neuroinflammation in PND are discussed.

## Microglia in the Brain

The glial population in the CNS consists of microglia, oligodendrocytes, and astrocytes (Standring, 2016). The microglia account for between 5 and 12% of the total number of cells in the brain (Lawson et al., 1990). Adult microglia derive from primitive myeloid precursors that arise in the yolk sac early during embryonic development, after which they self-maintain locally and independently of blood-borne myeloid precursors (Greter et al., 2015). As their name suggests, microglia have a small cell body with highly branched processes in normal physiological conditions. As the resident macrophage cells, they act as immune sentinels in the CNS to sustain normal brain functions under healthy conditions. It is shown that microglia can be rapidly activated in a large number of pathological conditions such as inflammation. The activated microglia in the CNS are commonly referred to as M1-like or M2-like (Mosser and Edwards, 2008; Martinez and Gordon, 2014). The M1 microglia originally act to an insult and promote a proinflammatory response, while the M2 microglia are involved in tissue repair and remodeling and exert anti-inflammatory effect (Safavynia and Goldstein, 2018). However, the simple M1/M2 categories are challenged because there are many overlapping phenotypes with various functions and activation pathways *in vivo* studies in disease models (Colton et al., 2006; Martinez and Gordon, 2014; Heppner et al., 2015; Amici et al., 2017). Mounting evidence suggests that microglia not only are simply the brain’s intrinsic immune cells but also are critical for neuronal circuit development, synaptic pruning, and brain homeostasis (Schafer et al., 2012; Zhan et al., 2014; Greter et al., 2015).

## Microglial Activation

Microglia are the principal immune cells of the brain. As mentioned above, they react to modifications in the cellular environment through a graded response, in which any induced changes in morphology or gene/protein expression from the homeostatic state are termed activation or reactivity (Lalancette-Hébert et al., 2012; Greenhalgh et al., 2020). One of the characteristics of the activation is morphological changes and increased numbers of microglia (the latter is referred to as microgliosis). For example, surgery induces the microglial phenotype to a reactive hypertrophic cell body and shortened processes in the hippocampal region of aged animals (Terrando et al., 2016; Zhang et al., 2019). On the other hand, aged animals displayed cognitive impairment and microgliosis in the CA1 hippocampal region following surgery (Hovens et al., 2013; Miller-Rhodes et al., 2019; Wang et al., 2019). Two possible mechanisms for microgliosis have been considered. First, it has been suggested that resident microglia proliferate (Inoue and Tsuda, 2018), but there is no study about microglia proliferation after anesthesia and surgery. Second, it has been proposed that bone-marrow-derived circulating monocytes may infiltrate into the hippocampus through the blood-brain barrier (BBB) and differentiate into microglia-like cells (Xu et al., 2014; Feng et al., 2017). Both microglia and astrocytes upregulate expression of chemokines such as monocyte chemoattractant protein 1 (MCP-1/CCL2), which further facilitate monocyte recruitment into the hippocampus under inflammatory conditions (Xu et al., 2017). It remains to be elucidated whether such microgliosis following surgery relies on the local expansion of mature microglia or is achieved by infiltrating monocytes of blood. One study proposed that the infiltrating myeloid cells do not persist in the CNS after inflammation resolution and thus do not contribute to resident microglia (Ajami et al., 2011).

Due to the shared lineage of microglia and macrophages, many markers are common to both cell types. Mature microglia, similar to blood monocytes and other tissue-resident macrophages, express common markers such as the integrin CD11b, ionized calcium-binding adapter molecule 1 (Iba1), fractalkine receptor CX3CR1, Csf-1R and CD68 (Vizcaychipi et al., 2011; Hovens et al., 2013; Qiu et al., 2016; Feng et al., 2017). Thus, the microglia detected by immunostaining have not been thoroughly distinguished by their derivation. High throughput gene expression studies might identify the genes distinguishing microglia from other cell types in the CNS or in the periphery (Tay et al., 2017), which have identified surface markers and transcription factors specifically expressed by steady-state microglia but not by other macrophage populations or monocytes. These microglia-specific markers include Fc receptor-like S, purinergic receptor P2YG protein-coupled 12, sialic acid-binding immunoglobulin-type lectin H, Tmem119 and Trem2 (Chiu et al., 2013; Butovsky et al., 2014; Bennett et al., 2016; Amici et al., 2017; Grassivaro et al., 2020). However, little is known about whether the microglia-specific surface markers and transcription factors alter their expression in neuroinflammation. Also, the molecular changes and functional difference between resident microglia and the monocyte-derived “microglia” remains ambiguous in PND.

Activated microglia are characterized by the changes in whole-genome expression and function in addition to morphological changes. Accumulating evidence indicates that anesthesia and surgery cause different degrees of microglial activation. The activation results in an inflammatory cascade promoting the synthesis and the secretion of inflammatory cytokines (IL-1β, IL-6, and TNF-α) and other inflammatory mediators (Buvanendran et al., 2006; Wang et al., 2019). Also, activated microglia recruit more blood monocytes (namely bone marrow-derived macrophages) into the CNS *via* reciprocal TNF-α expression (D’Mello et al., 2009). Neuroinflammation has become a key hallmark of neurological complications including PND (Spangenberg and Green, 2017; Subramaniyan and Terrando, 2019). The amplifying neuroinflammation and microglial activation could contribute to the development of PND (Hovens et al., 2013; Wang et al., 2015, 2016; Feng et al., 2017; Zhou X. et al., 2020). There is a limited amount of data about changes in microglia in the perioperative period in clinical studies. Non-invasive neuroimaging techniques may provide opportunities to assess the role of microglia directly (Tronel et al., 2017; Hierro-Bujalance et al., 2018). For example, microglial activation can be measured by positron emission tomography using uptake of [^11^C]PBR28, which binds to the translocator protein, a protein upregulated in activated microglia and astrocytes (Datta et al., 2017; Forsberg et al., 2017; Albrecht et al., 2019; Werry et al., 2019). A recent clinical study demonstrated that patients showed a global downregulation of gray matter [^11^C]PBR28 binding in the early postoperative period, recovering or even increasing after 3 months. These processes may be related to post-surgical impairments of cognitive function (Forsberg et al., 2017). Depletion of microglia or interrupting microglial activation in hippocampus suppresses neuroinflammation and/or cognitive decline after surgery (Wan et al., 2014; Kawano et al., 2015; Li et al., 2016, 2018; Wang et al., 2016; Feng et al., 2017; Zhang et al., 2019; Wen et al., 2020; Zhou Y. et al., 2020), providing evidence that microglia may play critical roles in neuroinflammation and PND.

## How the Microglia Are Activated

### Systemic Inflammation Induced by Surgical Trauma

Systemic inflammation can induce neuroinflammation and cognitive dysfunction in aged animals (Yamanaka et al., 2017; Huang et al., 2018). It is well-known that aseptic surgical trauma induces a systemic inflammatory response (Ni Choileain and Redmond, 2006). Damage-associated molecular patterns (DAMPs) are released by the damaged cells at the site of injury and promote and exacerbate the inflammatory response (Andersson and Tracey, 2011). Among the DAMPs, high mobility group box 1 (HMGB1) is the most studied as it has been described in preclinical models of cognitive impairment (Chavan et al., 2012; Li et al., 2013; Terrando et al., 2016). Increased levels of HMGB1 after surgery induce macrophage activation and the release of the pro-inflammatory cytokines (Terrando et al., 2016), which may induce an age-associated BBB dysfunction and increase its permeability (Yang et al., 2017). HMGB1 itself and these cytokines cross the BBB by diffusion or active transport causing macrophage migration into the hippocampus and microglia activation (Terrando et al., 2011, 2016). The activated microglia are the primary source of inflammatory cytokines that regulate microglia under feedback control (Hanisch, 2002). For example, following abdominal surgery under local anesthesia, the levels of TNF-α, IL-6, and microglia activation are increased (Xu et al., 2014). Splenectomy performed under general anesthesia triggers a cognitive decline that may associate with proinflammatory cytokine-dependent activation of glial cells in the hippocampus (Wan et al., 2007). The peripherally produced cytokines can trigger neuroinflammation by activating microglia (Terrando et al., 2011; Hirsch et al., 2016), resulting in direct neurotoxicity and a cognitive decline following surgery.

### General Anesthetics

The target organ of general anesthesia is the brain, but whether it is the main culprit causing cognitive decline by microglia remains controversial. The isoflurane or ketamine anesthesia causes morphological changes of microglia in rodents by using *in vivo* two-photon microscopy (Sun et al., 2019). It suggests that anesthetics may alter the function of microglia. Inhaled anesthetics have been demonstrated to cause neuroinflammation by activating microglia and may be involved in PND (Shen et al., 2013; Yan et al., 2016; Quiroz-Padilla et al., 2018; Wang et al., 2018). But a recent study showed that exposure to sevoflurane anesthesia for 8 h did not alter microglial activation in the adult monkey. The exposure had almost no effect on cognitive function (Walters et al., 2019). Etomidate, an intravenous anesthetic, induces PND attributed to hippocampal microglial activation during the early pathological stage (Li et al., 2020). But propofol, a widely used intravenous anesthetic, has no effects on neuroinflammation and cognition in the Alzheimer’s transgenic model (Mardini et al., 2017). Another study showed that propofol-induced postoperative depressive-like behaviors, which is attributed to the inhibition of microglial activation (Song et al., 2019). *in vitro* studies show propofol has neuroprotective effects by attenuating inflammatory response in microglia (Gui et al., 2012; Peng et al., 2014). Also, propofol and other anesthetics have been demonstrated to possess neuroprotective effects (Matchett et al., 2009; Fan et al., 2015). These conflicting findings may be due to the anesthetic agent, concentration, duration of the exposures, methodological variation, and so on. Different anesthetics may modulate immune signaling pathways through different manners and show anti-inflammatory and proinflammatory effects in neuroinflammation. The effects of anesthetics on microglial activation in PND remain to be determined. The *in vivo* imaging in awake and anesthetized animals could help study microglia-neuron interactions (Liu Y. U. et al., 2019).

### Additional Mechanisms

A recent study showed that peripheral surgery-induced CNS mast cell degranulation, which could trigger microglial activation and neuronal damage, contributing to PND (Zhang et al., 2016). Moreover, reactive oxygen species induced by nicotinamide adenine dinucleotide phosphate oxidase cause microglial activation that contributes to the neuroinflammation after the surgery (Qiu et al., 2016). Sirtuin-1 (SIRT1) is a member of the class III histone/protein deacetylase involved in the repression of inflammation (Kauppinen et al., 2013; Xie et al., 2013). SIRT1 activation inhibits nuclear factor kappa B signaling and enhances the resolution of inflammation (Kauppinen et al., 2013). Anesthesia and surgery inhibit hippocampal SIRT1 expression, resulting in microglial activation and an increase of proinflammatory cytokines in the hippocampus (Yan et al., 2019). Following tibial fracture surgery, the expression of CCL2 is upregulated in activated astrocytes. The astrocyte-derived CCL2 activates microglia participating in surgery-induced cognitive dysfunction and neuroinflammation (Xu et al., 2017).

## Activated Microglia Act on Neurons

Microglia mediate the inflammatory response in the hippocampus, resulting in the alteration of glutamatergic synaptic transmission and plasticity, which may underlie the behavioral comorbidities seen in patients (Riazi et al., 2015). The changes of inflammatory molecules in the brain following surgery and anesthesia may also directly bind receptors on neurons to alter neural actions and their normal adaptive roles. For example, TNF-α derived from glia mediates synaptic scaling *via* neuronal TNF receptors (Stellwagen and Malenka, 2006). A growing body of evidence indicates that in the aged brain, synaptic plasticity and memory show increased vulnerability to impairment by the IL-1β (Trompet et al., 2008; Patterson, 2015; Prieto et al., 2015). Other inflammatory mediators such as IL-6 and IL-9 are linked to physical and cognitive brain changes (McCarrey et al., 2014; Wharton et al., 2019). Electrophysiological recordings from CA1 hippocampal neurons revealed that PND mice exhibited impairment in AMPA receptor-mediated evoked excitatory postsynaptic currents (Wang et al., 2019). Astrocytes are the most abundant cell type in the CNS, which play a critical role in the formation and function of synapses. They also modulate neuronal excitability and plasticity (Greenhalgh et al., 2020). For example, hippocampal astrocyte dysfunction contributes to etomidate-induced long-lasting synaptic inhibition and cognitive dysfunction in older mice (Liu Y. et al., 2019). A subtype of reactive astrocytes, which are termed A1, is induced by activated microglia (Liddelow et al., 2017). A1 astrocytes contribute to the death of neurons and oligodendrocytes in neurodegenerative disorders (Liddelow et al., 2017). The A1-specific astrocyte activation is triggered by microglia during the initial pathological stage of PND and induces long-term synaptic inhibition and cognitive deficiencies (Li et al., 2020). Tau protein is primarily localized in CNS neurons and contributes to axonal integrity, whose tangles are strongly linked to neurodegeneration (Yang and Wang, 2018). The pathological mechanism of tau protein is associated with chronic neuroinflammatory processes, in which microglia play an important role (Vogels et al., 2019). The complement system is an important part of the innate immune system and involved in many neurological and neuropsychiatric diseases (Morgan, 2015). C3 levels and C3a receptor expression are specifically increased in the hippocampus after surgery. The C3a receptor activation contributes to neuroinflammation possibly through microglial activation, thereby resulting in the synaptic loss (Xiong et al., 2018). Taken together, these studies suggest that the neurons respond directly or indirectly to the inflammatory milieu induced by activated microglia and are influenced to affect cognitive changes. But the details of how activated microglia impair neurocognitive function after surgery warrants further research in the future.

## Why Are the Elderly More Vulnerable to PND?

As mentioned above, microglia play an important role in PND associated with neuroinflammation. PND is mainly seen in the elderly, and experimental studies also showed that it occurs frequently in old animals. Anesthesia and/or surgery did not cause a change in cognitive function in young adult mice (Zhao et al., 2016; Wang et al., 2018; Zhou X. et al., 2020). Hefendehl’s study showed that compared to young mice, aged microglia mice showed different levels of morphological changes (Hefendehl et al., 2014). Moreover, in the normal physiological state, aged microglia have higher expression of pro-inflammatory genes and antigen-presenting markers, while anti-inflammatory cytokines and microglial activation inhibitory factors are down-regulated (Mosher and Wyss-Coray, 2014). The proinflammatory cytokines such as TNF-α and IL-1β released from hippocampal microglia isolated from aged rats following stimulation with lipopolysaccharide was significantly higher in comparison with young rats (Kawano et al., 2015). The shift of aged microglia tends to the proinflammatory’ phenotypes (termed microglial priming) and may reflect an increase in inflammation associated with aging (Luo et al., 2010; Angelova and Brown, 2019). A recent study demonstrated that the hippocampal expression of SIRT1, which is associated with inflammation, decreased with age, resulting in microglial activation and increased proinflammatory cytokines in the hippocampus of aged rats. The trend of declining SIRT1 expression further deteriorated in aged rats after exposure to anesthesia and surgery (Yan et al., 2019). Moreover, the increased levels of NLRP3 expression in aged relative to young mice were observed in the hippocampus (Wang et al., 2018). The age-related morphological and functional changes in microglia may contribute to the susceptibility of the aging brain to dysfunction, often resulting in maladaptive responses, chronic inflammation, and worsened outcomes after injury (Koellhoffer et al., 2017). Whether microglia are the primary players in PND is worthy to study further.

## Conclusions

**Figure 1:**
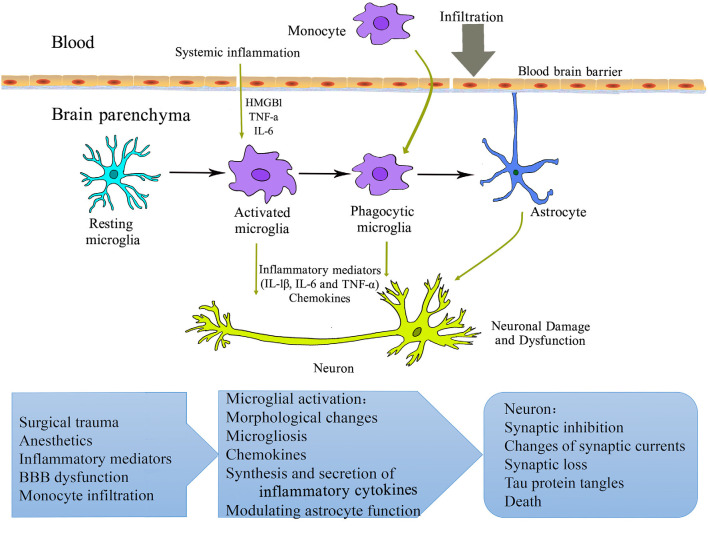
Schematic overview of microglial mechanisms involved in perioperative neurocognitive disorder (PND). Surgery and anesthesia induce a systemic inflammatory response. The increased pro-inflammatory mediators (cytokines, chemokines, alarmins, etc.) may compromise blood-brain barrier integrity, resulting in the infiltration of peripheral cells/factors into the brain parenchyma. Microglia are activated and initiate a cascade of inflammatory events that further activate other microglia and astrocyte. These processes contribute to neuronal damage and dysfunction and perioperative neurocognitive disorder. HMGB1, high-mobility group box 1 protein; IL, interleukin; TNF, tumor necrosis factor.

PND is a widespread phenomenon following the surgery and anesthesia and can have detrimental effects on an individual’s quality of life and well-being. The pathogenesis of PND is not fully understood. Activation of microglia and neuro-glial interactions seem to be key mechanisms in PND (Figure 1). The precise mechanisms of microglia in PND so far have not been clear. Animal models have suggested that cognitive dysfunction is owing to neuroinflammation microglia involved, but clinical studies have not provided definite evidence on microglia involved in PND. Moreover, there are no clinical trials targeting microglia to lessen PND. Hopefully, there have been new tools to extensively characterize and interrogate complex microglia-neurons interactions in development and neurodegenerative disease, which include the generation of microglia in brain organoids (Ormel et al., 2018; Verheijen, 2019), massive single-cell sequencing datasets of microglia in health and disease (Mathys et al., 2017; Haage et al., 2019; Masuda et al., 2019; Van Hove et al., 2019), and a 3D triculture system (Park et al., 2018). Clinically, the innovation of PET and other neuroimaging techniques will improve our understanding of the microglial mechanism in PND. A better understanding of the role of microglia in PND could be helpful to treat patients more effectively in the perioperative period and find strategies target to microglia to prevent and/or treat PND in the elderly conditions.

## Author Contributions

WF designed and drafted the manuscript and figure. LM and XZ analyzed the data. FH and HH revised the manuscript. All authors contributed to the article and approved the submitted version.

## Conflict of Interest

The authors declare that the research was conducted in the absence of any commercial or financial relationships that could be construed as a potential conflict of interest.
